# A specialized reciprocal connectivity suggests a link between the mechanisms by which the superior colliculus and parabigeminal nucleus produce defensive behaviors in rodents

**DOI:** 10.1038/s41598-020-72848-0

**Published:** 2020-10-01

**Authors:** Alfonso Deichler, Denisse Carrasco, Luciana Lopez-Jury, Tomas Vega-Zuniga, Natalia Márquez, Jorge Mpodozis, Gonzalo J. Marín

**Affiliations:** 1 Laboratorio de Neurobiología Y Biología del Conocer, Departamento de Biología, Facultad de Ciencias, Universidad de Chile, Las Palmeras, 3425 Santiago, Chile; 2 Institute of Science and Technology Austria (IST Austria), Klosterneuburg, Austria; 3 Facultad de Medicina, Universidad Finis Terrae, Santiago, Chile

**Keywords:** Neural circuits, Visual system

## Abstract

The parabigeminal nucleus (PBG) is the mammalian homologue to the isthmic complex of other vertebrates. Optogenetic stimulation of the PBG induces freezing and escape in mice, a result thought to be caused by a PBG projection to the central nucleus of the amygdala. However, the isthmic complex, including the PBG, has been classically considered satellite nuclei of the Superior Colliculus (SC), which upon stimulation of its medial part also triggers fear and avoidance reactions. As the PBG-SC connectivity is not well characterized, we investigated whether the topology of the PBG projection to the SC could be related to the behavioral consequences of PBG stimulation. To that end, we performed immunohistochemistry, in situ hybridization and neural tracer injections in the SC and PBG in a diurnal rodent, the *Octodon degus*. We found that all PBG neurons expressed both glutamatergic and cholinergic markers and were distributed in clearly defined anterior (aPBG) and posterior (pPBG) subdivisions. The pPBG is connected reciprocally and topographically to the ipsilateral SC, whereas the aPBG receives afferent axons from the ipsilateral SC and projected exclusively to the contralateral SC. This contralateral projection forms a dense field of terminals that is restricted to the medial SC, in correspondence with the SC representation of the aerial binocular field which, we also found, in *O. degus* prompted escape reactions upon looming stimulation. Therefore, this specialized topography allows binocular interactions in the SC region controlling responses to aerial predators, suggesting a link between the mechanisms by which the SC and PBG produce defensive behaviors.

## Introduction

Examining the connectome of a brain structure provides valuable insights into the neural mechanisms this structure implements and its possible contributions to behavior. This principle is especially evident in the superior colliculus (SC), a laminated mesencephalic center manifestly involved in several sensorimotor transformations. The SC integrates visual inputs in the superficial layers, from the retina, cortical and subcortical structures^1–3^, with somatosensory and auditory inputs in the intermediate layers, forming a multisensory map of space in register with a motor map in the deep layers^1,2,4^. This cross modal, topographic organization is thought to be the basis for rapid sensorimotor coordinations, such as orientating eyes and head towards salient stimuli^5^, and freezing and escape in the presence of aversive stimuli^1,6^.

In rodents, these opposite behaviors depend on what portion of the SC is being stimulated. Electrical and optogenetic activation of the medial SC, representing the overhead visual field, elicit freezing and escape reactions, while stimulation of the lateral SC, representing the lower visual field, evokes exploratory behaviors^1,7^. Likewise, behavioral studies in mice have shown that moving and looming stimuli trigger freezing and avoidance if presented in the overhead visual field^8–10^, while moving food items, such as crickets, trigger orienting and capture maneuvers if presented in the lower visual field^11^.

In support of these results, there is a marked segregation of the afferent and efferent connections in the SC of rodents that reveals its specialized medial–lateral organization^12,13^. For instance, W3 retinal ganglion cells (RGCs), the major retinal afference to the SC of mice, display a denser distribution in the ventral retina (dorsal visual field, projecting to the medial SC), and respond selectively to moving objects simulating an aerial predator^14^, while alpha-RGCs, an SC projecting RGC type involved in object recognition, are denser in the dorsal temporal retina^15^ (lower-frontal visual field, projecting to lateral and rostral SC). Also, at the motor level, the crossed tecto-bulbar and uncrossed tecto-pontine pathways—the major descending pathways controlling approach and defense behaviors—originate, preferentially, from the deep layers of the lateral and medial SC, respectively^1^.

In view of this evidence, it should be expected that the spatial connectivity pattern established by the SC with other structures indicates the structures' involvement in approach or avoidance, especially if that connectivity is differentially biased in the medial–lateral collicular framework^12,13^. The parabigeminal nucleus (PBG), an isthmic nucleus massively connected with the superficial SC layers, may represent a case in point. Optogenetic stimulation of PBG-projecting collicular neurons and of PBG neurons themselves produces escape behaviors in mice^16,17^. These responses were attributed to PBG projections to the central nucleus of the amygdala (CeA), a center prominently involved in fear and defensive reactions. However, this conclusion may overlook the possibility that the PBG's role in avoidance may also be linked to a direct modulation of SC-mediated defensive responses via a specialized PBG-SC connectivity.

In the present study, we characterized the PBG neurochemistry and connectivity pattern between the PBG and the SC in the diurnal rodent *Octodon degus*, especially assessing the extent to which these connections conform to the medial–lateral specificity of the SC. In addition, we described the escape behavior in response to visual stimuli in this animal, with a particular emphasis on how the organization of this aversive behavior conforms to the organization of the visual field and the anatomy of the parabigemino-tectal circuit. Our results suggest that the mechanisms by which the SC and the PBG produce defensive behaviors are closely linked by a specialized topography connecting both structures.

## Results

### Cytoarchitecture and immunohistochemistry of the PBG

**Figure 1:**
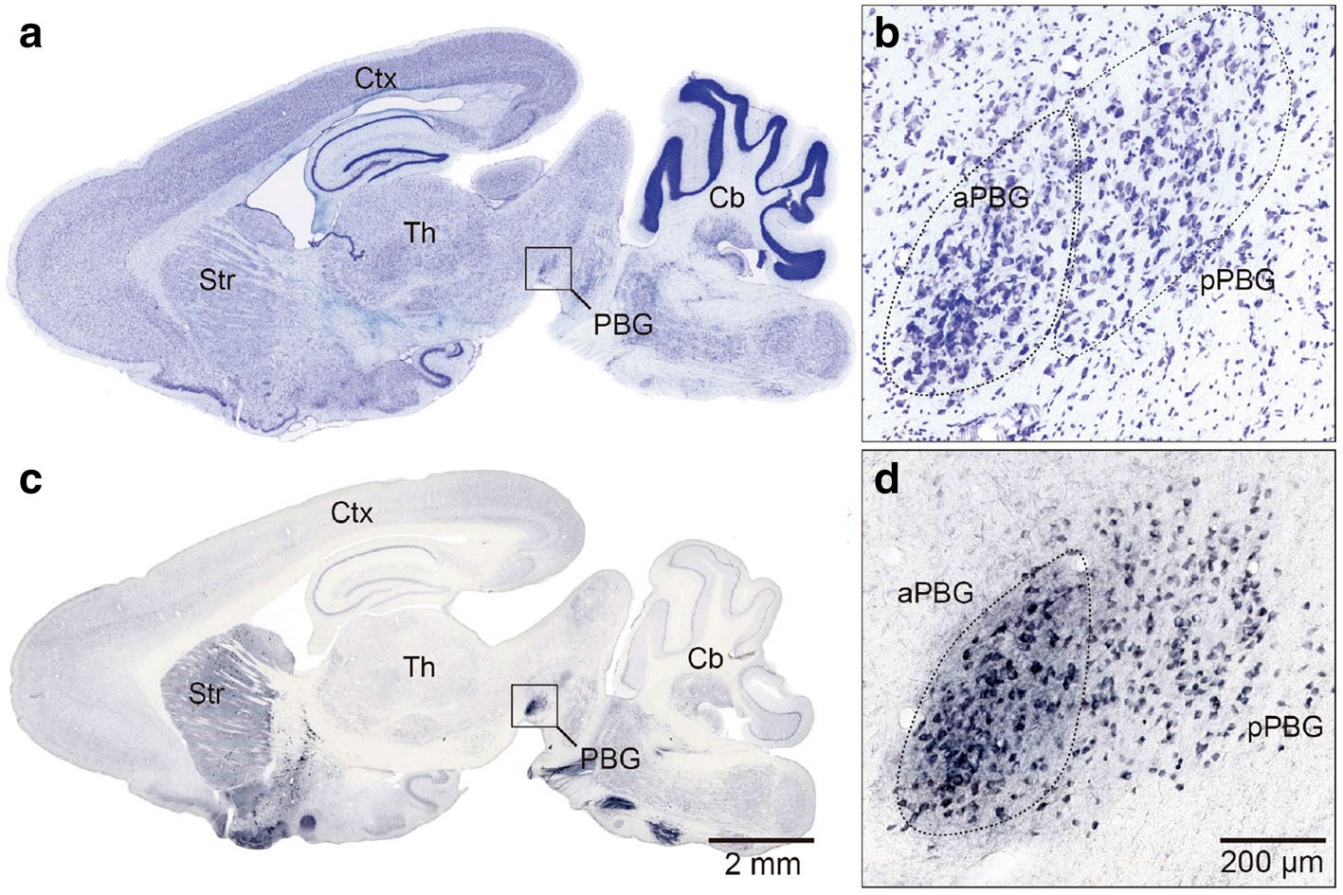
Cytoarchitectonic organization and ChAT immunoreactivity of the parabigeminal nucleus in the *O. degus*. Photomicrographs of Nissl stained (**a**, **b**) and ChAT immunoreacted (**c**, **d**) sagittal sections at the level of the PBG nucleus. At high magnification, two PBG divisions are clearly discerned; the anterior division (aPBG) showed larger somas and more intense labeling than the posterior division (pPBG) in both in Nissl (**b**) and ChAT (**d**) preparations. Cerebellum (Cb), cerebral cortex (Ctx), parabigeminal nucleus (PBG), striatum (Str), thalamus (Th).

In sagittal Nissl stained sections, the PBG appears as a band of densely packed cells lying in the lateral wall of the isthmic tegmentum (Fig. 1a,b). Comparative studies have shown that expression of the enzyme choline acetyltransferase (ChAT) is a characteristic of the isthmic nuclei projecting to the optic tectum or to the SC in all vertebrates^18^. Therefore immunohistochemistry (IHC) assays were performed for ChAT in sagittal brain slices of adult degus. The results showed that the PBG is readily identifiable by its intense ChAT-like immunoreactivity (Fig. 1c,d). These preparations also revealed that the PBG possesses two subdivisions arranged in the antero-posterior axis of the nucleus. In the anterior division (aPBG), the cells are darkly stained and densely packed. The posterior PBG (pPBG) contains less stained and loosely aggregated neurons than the aPBG (Fig. 1b,c).

### In situ hybridization for glutamatergic and cholinergic markers in the PBG

Studies in birds have shown that neurons of the avian Ipc, the presumptive homologue of the PBG, are rich in the expression of vesicular glutamate transporter 2 (VGluT2) mRNA^19,20^. To evaluate a possible glutamatergic identity of the PBG, we performed in situ hybridization assays using VGluT2 mRNA probes.

**Figure 2:**
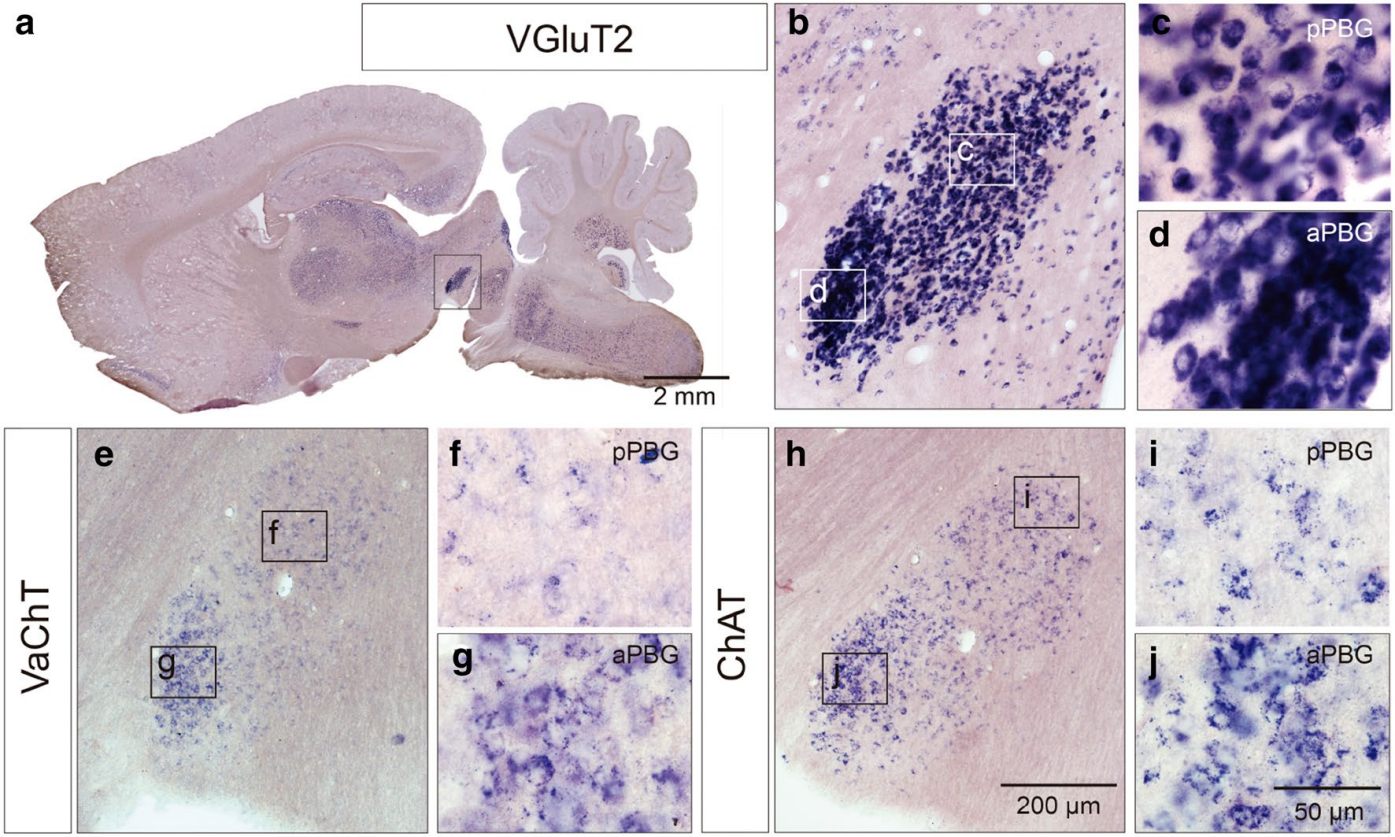
Expression of glutamatergic and cholinergic markers in the PBG. Sagittal sections showing in situ hybridizations for the vesicular glutamate transporter 2 (VGluT2; **a**–**d**), vesicular acetylcholine transporter (VAChT; **e**–**g**) and choline acetyltransferase (ChAT; **h**–**j**) mRNAs. Both PBG subdivisions show expression for all markers and VGluT2 mRNA shows the highest label intensity (**a**–**d**).

In all cases tested (*n* = 9), we found a strong expression of VGluT2 mRNA in neurons of both subdivisions of the PBG (Fig. 2a–d). As observed in the Nissl and ChAT IHC preparations, the somata of the aPBG are both more intensely stained than the cells of the posterior subdivision. To reassess the cholinergic identity of the nucleus, we also performed in situ hybridization assays using VAChT and ChAT mRNA probes. In all cases, the PBG neurons of both divisions exhibited strong labelling for the respective probe (Fig. 2e–j).

**Figure 3:**
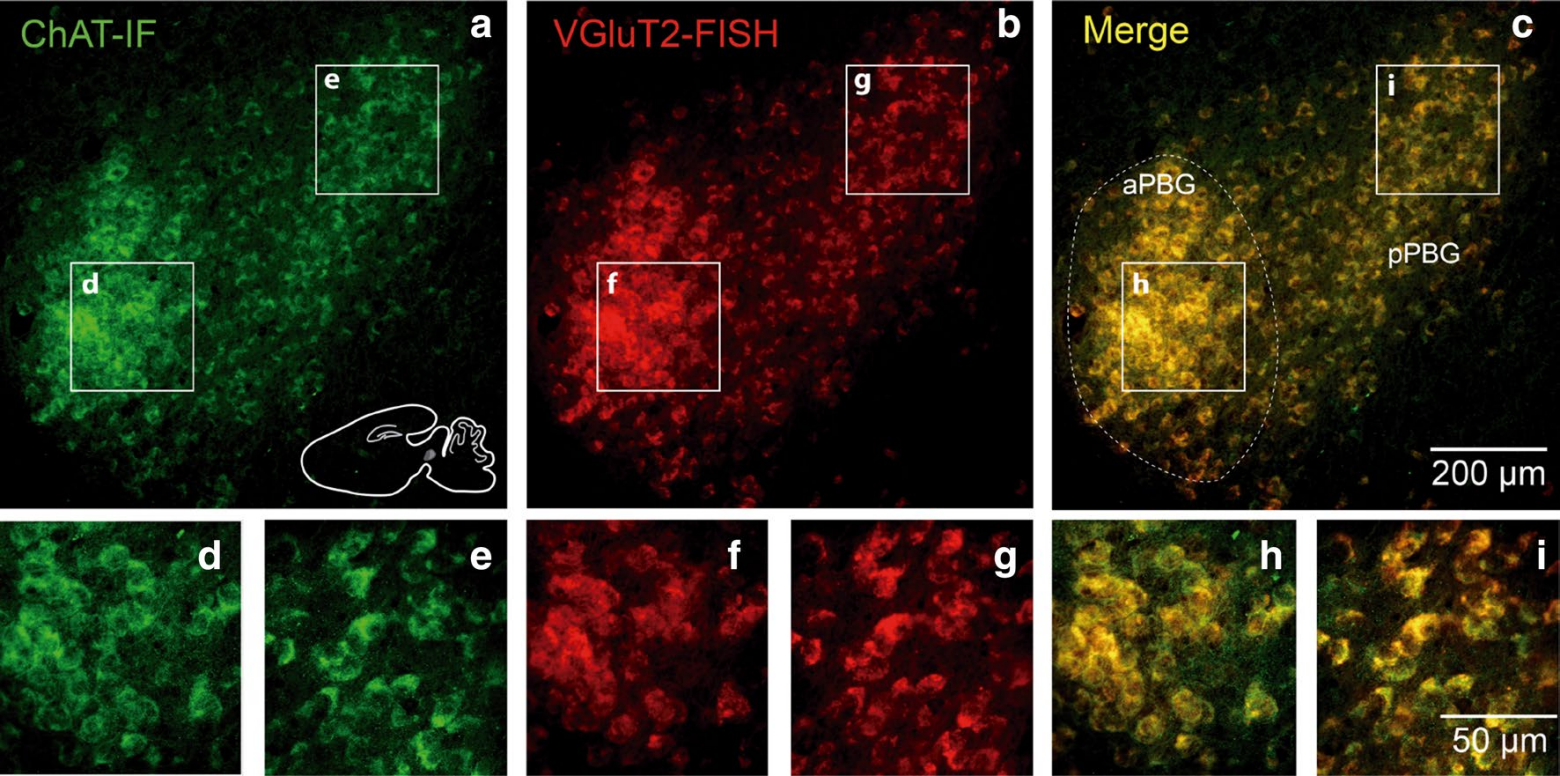
Co-expression of glutamatergic and cholinergic markers in the PBG neurons. Combined ChATIF and VGluT2 FISH show that individual PBG neurons co-express glutamatergic and cholinergic markers. Co-localization of the signal for ChAT and VGluT2 is present in both subdivision of the PBG.

Next, we investigated whether the ChAT and VGluT2 markers were expressed in the same or in different neurons. To that end, we performed immunofluorescence (IF) for ChAT in conjunction with fluorescent in situ hybridization (FISH) for VGluT2 in the PBG (*n* = 3). We found double labeling in all the cells in both subdivisions, indicating that all PBG neurons express both neurochemical markers (Fig. 3).

### Retrograde neuronal tracing of the PBG efferences to the SC

**Figure 4:**
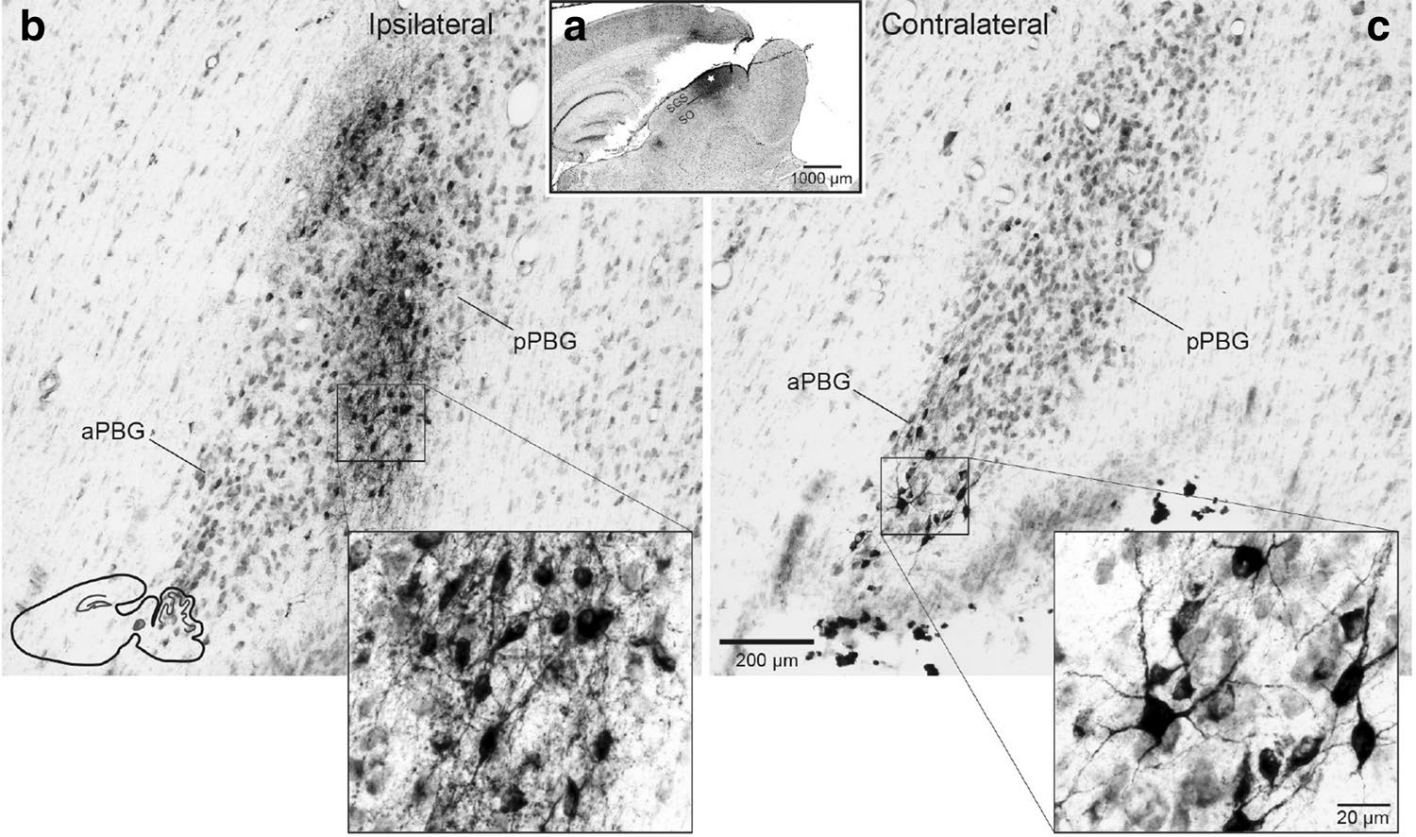
Bilateral parabigemino-tectal projections. Sagittal sections showing bilateral retrograde labeling in the PBG after a large CTB injection into the superficial SC (**a**). Note the difference in distribution and soma size of ipsi- and contralaterally projecting cells, with smaller labeled cells distributed in the ipsilateral pPBG division (**b**) and larger labeled cells clustered in the contralateral aPBG (**c**). Differences in cellular estimated volume were statistically significant (contra: 2294.5 ± 73.2 μm^3^ vs. ipsi: 984.6 ± 29.6 μm^3^ (mean ± SEM), *p* < 0.001. See text).

To characterize the hodological organization of the isthmo-tectal circuit in the degus we performed stereotaxic injections of the neural tracer cholera toxin subunit b (CTB) in the SC and PBG (*n* = 10). First, we performed large injections to cover most of the medio-lateral extent of the collicular surface. In agreement with previous studies in other mammals^21–29^, we found that the projection from the PBG to the SC is bilateral, as retrogradely labelled cells appeared in the PBGs ipsi- and contralateral to the injection site (Fig. 4). A closer inspection of sagittal sections revealed that the ipsi- and contralaterally projecting PBG neurons corresponded, respectively, to the posterior and anterior PBG subdivisions. Cells projecting to the contralateral SC were located in the aPBG (Fig. 4c), whereas cells projecting to the ipsilateral SC were restricted to the posterior PBG (Fig. 4b). In addition, labeled cells in the contralateral aPBG had significantly larger estimated somatic volume (2294.5 ± 73.2 μm^3^, mean ± SEM) than cells found in the ipsilateral pPBG (984.6 ± 29.6 μm^3^) (repeated measure ANOVA: *F*_*[1, 234]*_ = 153.1, *p* < 0.001). Thus, the PBG appears to be constituted by two neuronal classes that are discernible on the basis of cytoarchitectonic and hodological criteria.

**Figure 5:**
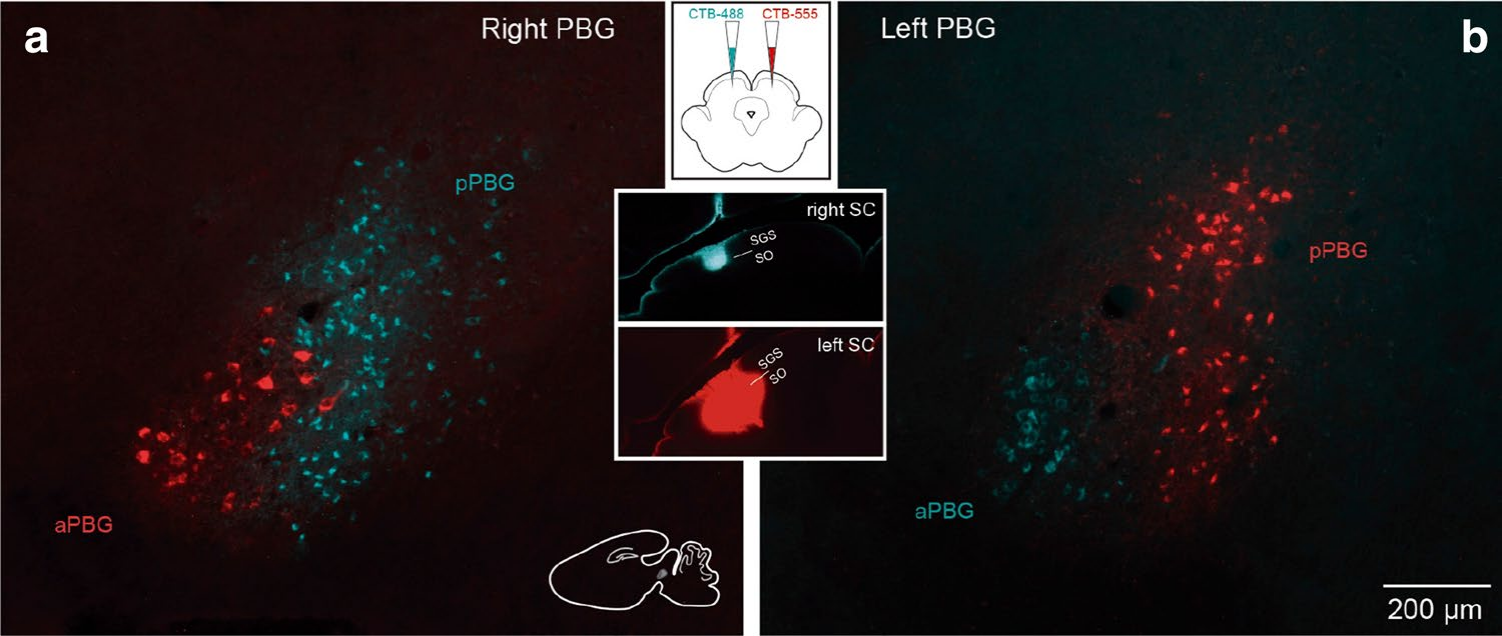
Ipsilateral and contralateral parabigemino-tectal neurons constitute separate neural populations. Sagittal sections depicting the retrograde tracing transport after injections of Alexa Fluor-conjugated CTB into near homotopic loci of each tectal hemisphere. Note that the retrograde labeled cells segregate into each PBG division according to the tectal side they project to, with no double-labeled cells, indicating that pPBG and aPBG cells project exclusively to the ipsilateral and contralateral SC, respectively. Insets: schematic of the experiment and injection sites.

To obtain a better picture of the segregation of these two neuronal classes in the respective PBG domains, we performed double injections of fluorescent CTB (CTB-Alexa Fluor 488 and CTB-Alexa Fluor 555) into near homotopic loci of each SC. Our results showed that the ipsi- and contralaterally projecting neurons were almost completely segregated, with some minor overlap in the zone of transition between the aPBG and pPBG. However, even in that zone, we did not find any double-labeled cell, demonstrating that the ipsilateral and contralateral PBG-tectal projections originate from separated neuronal populations (Fig. 5).

### Medial–lateral organization of the PBG projection upon the tectum

We next evaluated whether the PBG-SC projection follows a medial–lateral organization in the SC. In this set of experiments, we performed small CTB injections into either the medial or lateral portions of the SC (*n* = 4). In both types of experiments, the retrogradely labeled neurons in the PBG ipsilateral to the injection site were restricted to the posterior domain of the nucleus. The location of these neurons varied according to the position of the injection, indicating that the ipsilateral projection is topographic. On the other hand, retrogradely labeled neurons in the PBG contralateral to the injection site were only found after tracer injections in the medial portion of the SC, and these neurons were restricted to the anterior domain of the PBG.

**Figure 6:**
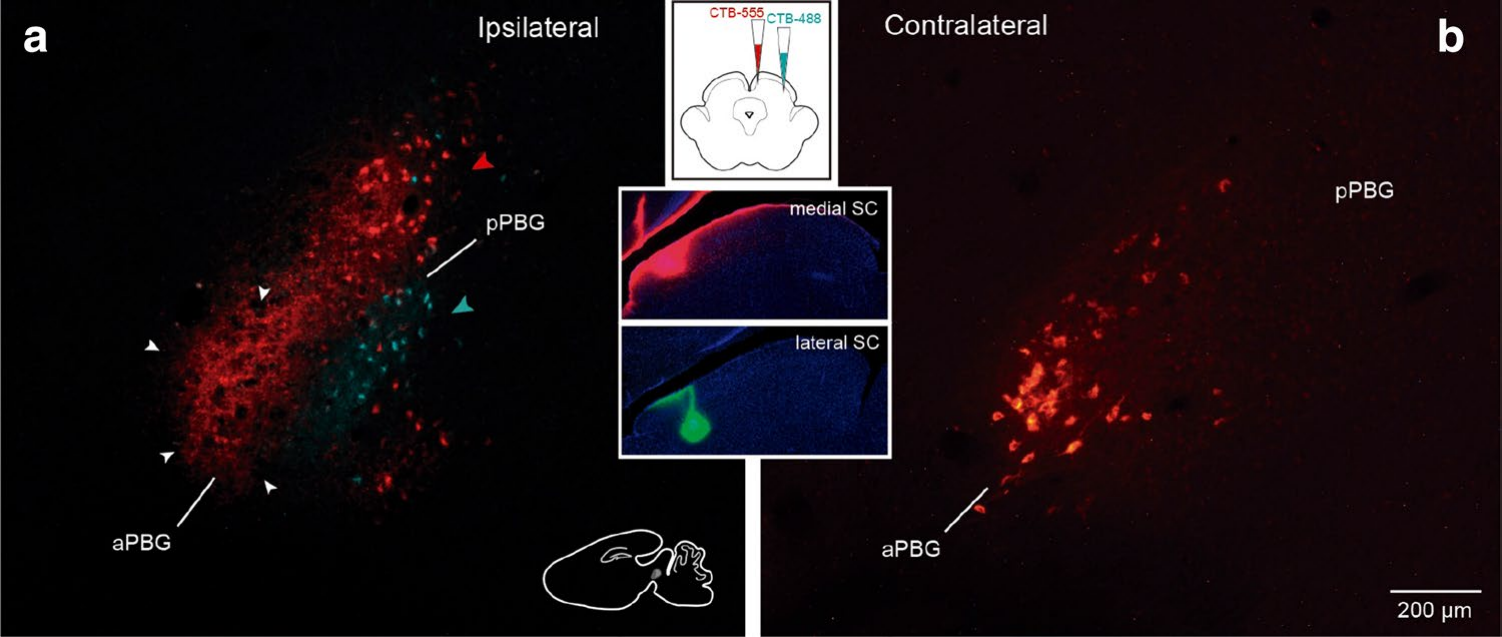
Medial–lateral topography of the PBG-SC projection. Sagittal sections showing the distribution of retrogradely labeled cells in the PBG after a double injection of fluorescent CTB probes into the medial (CTB-555) and lateral (CTB-488) superficial SC. In the ipsilateral pPBG, cells filled by either fluorophore are segregated in space indicating that this division projects topographically to the SC (green and red arrowheads in (**a**)); terminal fibers filled only with the tracer injected in the medial SC (CTB-555) are seen in the ipsilateral aPBG (white arrowheads in (**a**)). In the contralateral aPBG, retrograde transport is restricted to fluorescent CTB injected in the medial SC (**b**), indicating that this PBG division projects only to this part of the contralateral SC. Insets: schematic of the experiment and injection sites.

To confirm this segregation, we simultaneously injected CTB-555 in the medial part of the SC and CTB-488 in the lateral part of the same SC in three animals. As a result, in the ipsilateral PBG we found two clusters of labeled neurons, reflecting the topographic organization of the ipsilateral PBG-SC projection: CTB-555-labeled neurons were found in the dorsal posterior part of the pPBG and CTB-488-labeled neurons in the ventral anterior part of the pPBG (Fig. 6a). In the contralateral aPBG, cells were only labeled by CTB-555, reflecting that neurons of this division project exclusively to the medial part of the contralateral SC (Fig. 6b). In addition, the ipsilateral aPBG is replete with terminal fibers that are filled only by the tracer injected in the medial portion of the SC (Fig. 6a; S1).

**Figure 7:**
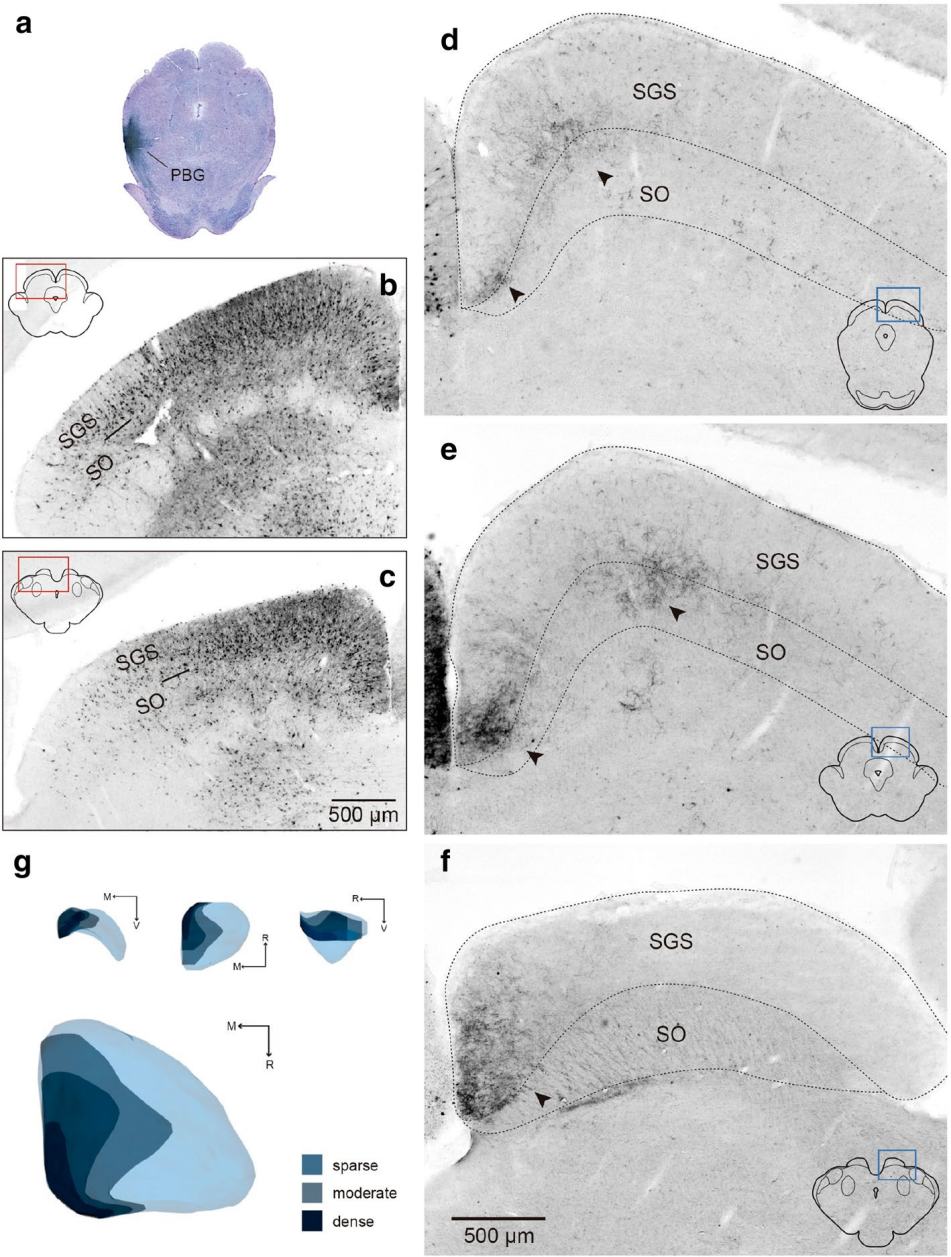
Topography of PBG axonal terminals in the superficial SC. Coronal sections showing the distribution of retrogradely labeled cells in the ipsilateral SC (**b**, **c**) and anterogradely labeled terminal field in the superficial contralateral SC (**d**–**f**) after a CTB injection in the PBG (**a**). Brain sections are arranged from caudal to rostral. Note that the PBG terminals are distributed in the medial aspect of the SC (arrowheads in **d**–**f**). Panel (**g**) displays a 3D reconstruction of the area occupied by the PBG fibers in the contralateral SC. Color represents qualitatively the relative abundance of labeled fibers and terminals after the PBG injection. Stratum griseum superficiale (SGS), stratum opticum (SO).

To describe the axon terminal field of the contralateral parabigemino-tectal projection, we injected CTB in the PBG (*n* = 2; Fig. 7a). In the ipsilateral SC, labeled cells were found in the superficial layers of the entire SC (Fig. 7b,c), similar to what has been shown in mice^17^. In the contralateral SC, the terminal fibers were restricted to the medial part of the SC (mSC), throughout the rostral-caudal axis, consistent with the retrograde results (Fig. 7d–g). In addition, in cases that we failed to target the PBG, but our tracer deposits were located in the medial-adjacent peri-parabigeminal area (*n* = 4) (also known as nucleus sagulum in the mouse, and microcellular tegmental nucleus in the rat^30^), we observed intense bilateral fiber labeling in the intermediate and deep collicular layers and in the dorso-lateral periaqueductal gray, as previously described in the cat^31^. This result reinforces our retrograde and anterograde experiments, confirming that the only source of projections to the superficial SC layers from this part of the isthmic tegmentum is the PBG.

**Figure 8:**
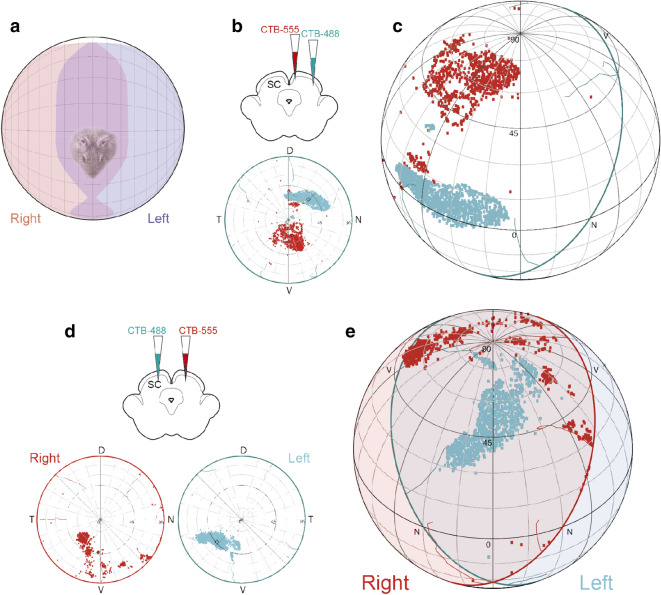
Organization of the degus visual field and its representation in the SC. (**a**) Upper view of an orthographic projection of the monocular and binocular visual field of the degus (data from Vega-zuniga et al.^45^). (**b**) Retinal reconstruction with labeled RGCs resulting from the CTB injections in the medial and lateral parts of the contralateral SC depicted in Fig. 6; location of the cuts and tears in the retina are displayed (cyan lines in (**b**) and left retina in (**d**) and red lines for the right retina in (**d**)) (**c**) Orthographic projection of the labeled RGCs into visual space. Labeled RGCs in the ventral retina (red), resulting from the medial SC injection, project to the upper binocular space. (**d**) Retinal reconstructions with labeled RGCs after two CTB injections in the medial SC depicted in Fig. 5. Note that in both cases the cells are located in the ventral retina, which corresponds to the upper binocular field as shown in (**e**).

Notably, the longitudinal zone defined by the contralateral parabigemino-tectal projection conforms to the representation of the binocular region in the upper visual field in the *O. degus*^32^ (Fig. 8). The ventral retina that looks to this aerial binocular field seems to lack ipsilateral projections to the medial SC, as shown by the very sparse labeling in the ipsilateral SC after monocular CTB injections (Fig. S2)^32^. Thus, the contralateral PBG projection provides the main ipsilateral retinal contribution to the SC representation of the aerial binocular field of *O. degus*. In support of this conclusion, injections in the mSC that labeled cells in the contralateral aPBG, also labeled RGCs in the ventral portion of the contralateral but not of the ipsilateral retina (Fig. S3). This contralateral retinal area was found to project to the binocular space of the upper visual field, after being orthographically projected into the visual field (Fig. 8b–e).

### Correspondence of the visual anatomy and the organization of escape behavior in the *Octodon degus*

**Figure 9:**
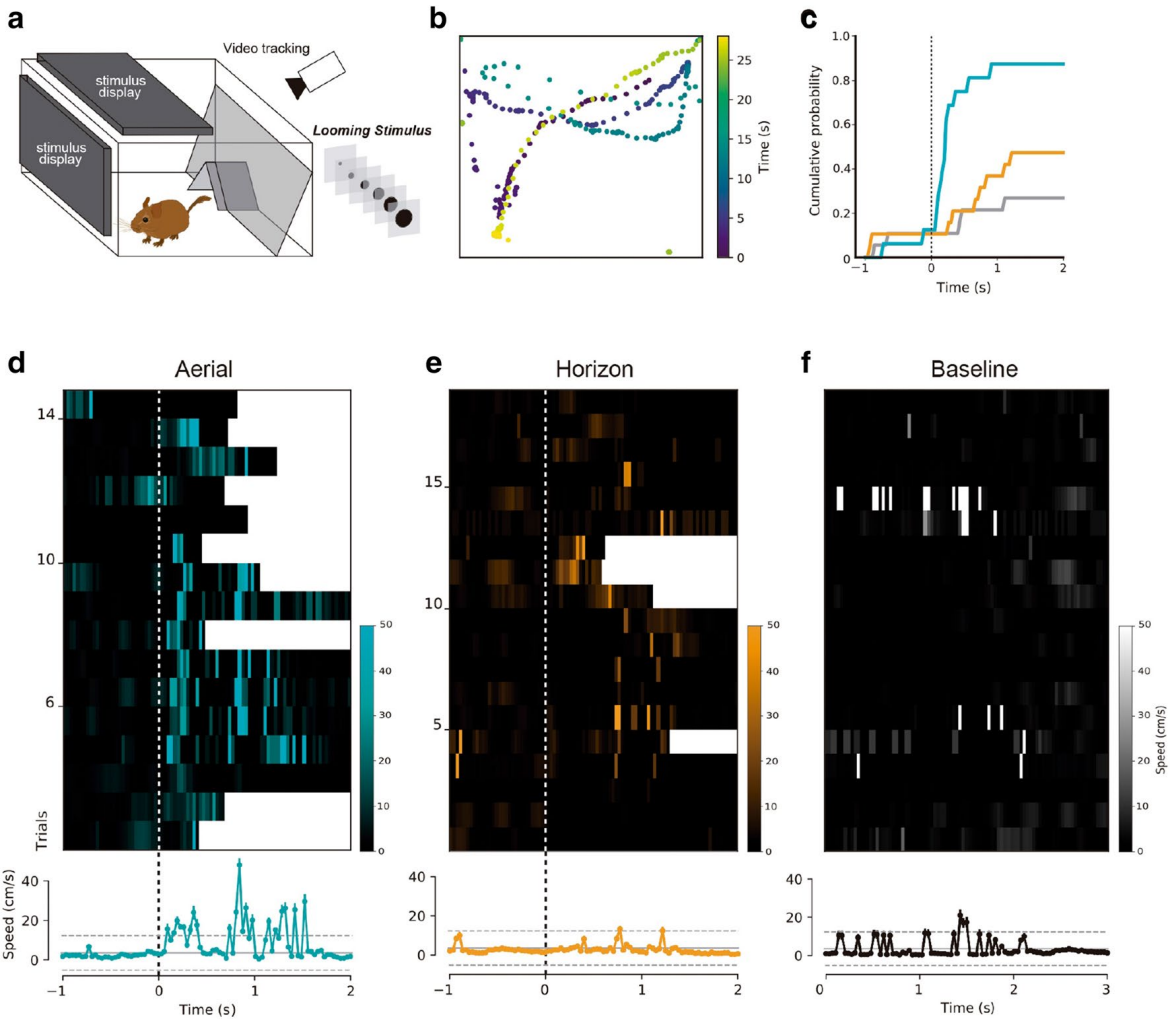
Dependence of escape behavior on stimulus location. (**a**) Schematic representation of the experimental set up. An expanding disc simulating a looming stimulus is presented either in the lower (horizon) or upper (aerial) monitor. (**b**) Example of a tracked trajectory of an *O. degus* in one trial. (**c**) Cumulative probability of having observed an escape response (defined as episodes where the degus turned to the refuge at speeds larger than 30 cm/s) over time for aerial stimulation (cyan line), horizon stimulation (brown line) and baseline (grey line). (**d**, **e**) Raster plots of movement speed in each trial for stimuli presented in the aerial (**d**) and horizontal (**e**) monitors (each row represents the first trial in different animals). (**f**) Raster plot of baseline movement without stimulation. Speed scales are presented beside each raster plot; in (**d**) and (**e**) black indicates no motion; white indicates entrance to the refuge. In (**f**) black indicates no motion; white indicates speeds > 50 cm/s; no entrance to the refuge occurred in these periods. Average velocity is displayed below each raster plot (mean ± SEM). Horizontal dashed lines indicate mean and the 95% confidence interval of movement speeds in the absence of visual stimuli. Time zero indicates the beginning of the stimulus.

Finally, in order to further show the correspondence between the organization of the visual field, parabigeminal projections and avoidance behavior across rodent species, we replicated in *O. degus* the same behavioral test used to investigate the escape response bias in mice and rats to aerial threatening stimuli^8–10^ (Fig. 9a,b). Individuals placed in an arena reliably flee to their refuge in response to the presentation of a looming stimulus in the overhead field of view. The proportion of escape reactions was significantly higher in response to “aerial” looming stimulation (10 out of 16 trials, 1 trial per animal; Fig. 9d), compared to when the same stimulus was presented on the frontal and lateral visual field (4 out of 19 trials; Fisher`s Exact test, *p* = 0.01534). To quantify and compare the responses to “aerial” versus “horizon” stimuli, escape was defined as episodes where the degus rapidly turned to the refuge at speeds exceeding 30 cm/s. The probability of observing an escape event was significantly larger when the looming stimulus was presented in the aerial visual field than in the lower visual field (Fig. 9c). Likewise, the increments in average velocity were larger in response to aerial than horizontal stimulation (Fig. 9d–f).

These results show that in *O. degus*, as in murine rodents, there is a strong bias towards avoidance behaviors when threatening moving stimuli are presented in the aerial binocular field, revealing the conservative character of the organization of visual anatomy and behavior across rodent species.

## Discussion

**Figure 10:**
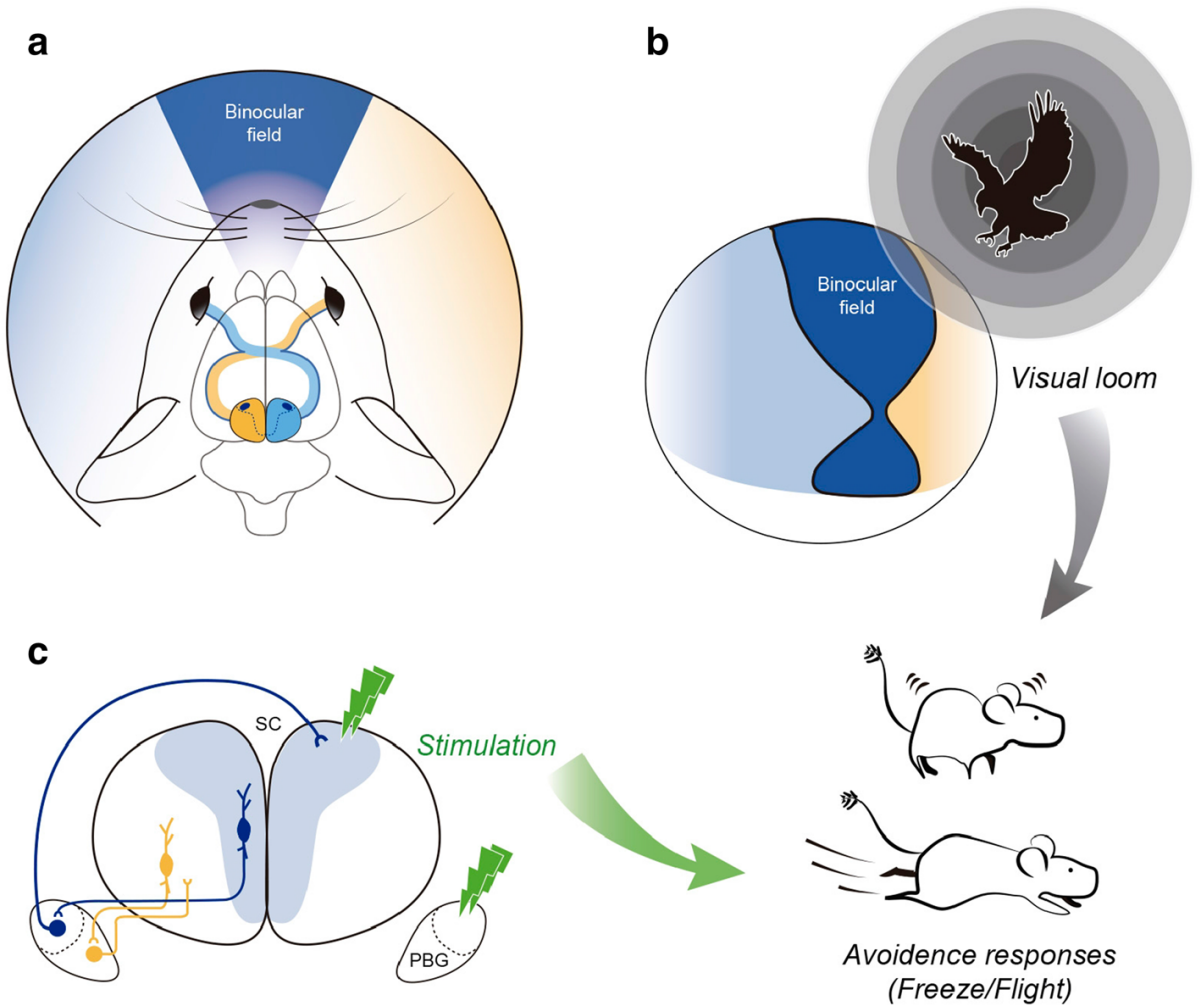
Proposed functional organization of the parabigemino-tectal circuit. (**a**) Schematic representing the largely crossed retino-collicular projection in *O. degus*. (**c**) Hodological relations between the parabigeminal nucleus and the superior colliculus in relation to the representation of binocular visual specializations and visually guided avoidance behaviors. The medial SC, representing the aerial binocular visual field (**b**), projects to the aPBG, which in turn, projects to the contralateral medial SC, allowing binocular operations in this part of the SC. Previous and present behavioral results indicate that in rodents the upper binocular field is involved in aerial anti-predatory vigilance (**b**)^8–10^. Stimulation of the medial SC and PBG also triggers avoidance responses (**c**)^1,7,16,17^. In this way, the contralateral PBG-SC projection could facilitate binocular integration and modulate upstream and downstream SC pathways that trigger fast defensive responses.

Our neural tracing results revealed that the PBG has two subdivisions, which are discernible on the basis of their cytoarchitecture and their pattern of connections with the SC. Neurons in the posterior subdivision project strictly to the ipsilateral SC, whereas neurons in the anterior subdivision project exclusively to the contralateral SC. Cells from both subdivisions co-express neurochemical makers associated to the release of glutamate and acetylcholine, suggesting a complex synaptic effect upon their neuronal targets. Recent reports in transgenic mouse lines also show co-expression of glutamatergic and cholinergic markers in the murine PBG^33,34^. The crossed PBG-SC projection defines a medial binocular area in the SC corresponding to the representation of the overhead visual field that previous studies in murines have shown to elicit escape and freezing upon visual stimulation^8–10^, a behavioral result that we now replicated in *O. degus*. In further support of the conservative character of the functional organization of parabigemino-tectal projections in rodents, preliminary experiments from our laboratory show that the PBG in the mouse display a similar hodological organization and topography of tectal projections as we have shown here in *O. degus* (Figs. S4 and S5). Also, data from the Allen Mouse Brain Atlas show that viral injections in the PBG and the adjacent nucleus sagulum produce a bilateral labeling of fibers that includes a longitudinal band of terminals in the medial part of the superficial layers of the contralateral SC, similar to what we describe in the *O. degus* and mice. The labeling observed in the intermediate and deep SC and the periaqueductal gray is consistent with projections from the nucleus sagulum^31^ (viral injections are freely available at the Mouse Brain Connectivity Atlas: https://connectivity.brainmap.org/?searchMode=source&sourceDomain=874&primaryStructureOnly=false&isi=false). Altogether, these results suggest that the rodent PBG can influence fast innate defensive reactions, such as freeze or flight, by direct connections to the SC (Fig. 10) in addition to the alternative route via connections to the amygdala.

### Organization of the Isthmo-tectal projections in mammals

Our results are in agreement with several studies in mammals reporting bilateral parabigemino-tectal projections^21–29^. Previous studies in rats^21,29^ and hamsters^24^, distinguished three components in the PBG, a dorsal and ventral divisions connected reciprocally with the ipsilateral SC, and a medial division connected contralaterally with the SC. However, as these observations were based on coronal sections, which in our experience make it difficult to differentiate divisions that are disposed in the sagittal plane, we suggest that the dorsal and ventral divisions reported in rats and hamsters both correspond to the pPBG of *O. degus*, and the medial division to the aPBG. In the same vein, Jiang et al.^25^ reported in the ferret that the PBG neurons projecting to the contralateral SC are located in the rostral PBG, while neurons projecting to the ipsilateral SC are located more caudally in the nucleus. Thus, a rostro-caudal parcellation of two hodological cell types seems to be a common characteristic of the mammalian PBG. Primates might be a possible exception, as Baizer et al.^22^ showed in *Macaca mulatta* and *M. fascicularis* that the anterior part of PBG generates both ipsi- and contralateral PBG-SC projections. However, these authors also pointed out that the contralateral projection originates from neurons of larger somata, indicating that two hodological neuronal types are also present in the primate PBG. The similarities in the organization of isthmic projections among mammals strongly suggest a conserved functional role for the PBG. In all species studied, the PBG generates specialized topographies that can be related to the representation of visual field and retinal specializations in the superior colliculus (see below).

### Functional implications

Based on optogenetic manipulations it has been suggested that the PBG generates escape reactions through glutamatergic connections with the CeA^16,17^. While a PBG-CeA projection has been reported in several studies^16,17,30,35^, this interpretation overlooks the tight reciprocal SC-PBG connections and the role of the SC in organizing escape reactions. Indeed, several studies have demonstrated that the SC is directly involved in the generation of antagonistic sensory-guided orienting responses, such as eye and head movements toward the stimulus or escape maneuvers away from it. In rodents, it has been shown that the selection and initiation of such responses depends on the region of the SC being stimulated, in a manner consistent with the organization of the visual field and visual ecology of this group of mammals. Namely, stimulation of the medial SC, where the upper visual field is represented, triggers avoidance responses such as freeze and escape, while stimulation of the lateral SC, where the lower visual field is mapped, induces exploratory behaviors^1,7,12^.

Visual defensive responses have a strong innate character, as escape and freezing are highly stereotyped and may occur without previous experience of the aversive stimuli^10^. The intermediate and deep layers of the SC participate in these responses through descending uncrossed projections to the dorsal peri-aqueductal gray (dPAG) and cuneiform nucleus^1,36^. It has been demonstrated that escape responses depend on activity propagation from the deep layers of the medial SC (dmSC) to the dPAG, the activation of which is critical to elicit responses to threat^37^. Excitatory dmSC neurons seem to represent the saliency of threatening stimuli, and a weak synaptic connection between dmSC and PAG neurons imposes a threshold requiring high dmSC neuronal firing to elicit escape^37^. Activity of looming sensitive neurons from the SC superficial layers, representing the approaching direction and time to collision with an incoming aerial predator, could be transmitted to the dmSC and trigger escape. It has been shown that inactivation of dPAG neurons switches looming-elicited responses from escape to freezing^37^.

On the other hand, the superficial SC layers may also contribute to defensive behaviors through more indirect ascending pathways. One of them connects collicular neurons of the stratum opticum with the caudal caudate/putamen division, via the rostro-lateral pulvinar (PulR)^38^. Another connects collicular wide-field tectal ganglion cells (TGCs) to the lateral amygdala (LA), via a bilateral diffuse projection to the caudal-medial division of the pulvinar (PulC)^7,38–40^. This pulvinar subdivision also projects to the temporal posterior cortex (TP)^41–43^, which then projects to the amygdala^43–46^. Fredes et al.^39^ noticed that TGCs projecting to the PulC increase significantly in number toward the medial aspect of the SC in the squirrel, whereas cells that project topographically to the PulR are homogenously distributed^39,42^. The matching topographies of tectofugal cells and the PBG-SC projections suggest a specific relation between the aPBG and the TCGs-PulC pathway and between the pPBG and the SC-PulR pathway, a possibility that needs further assessment.

While the role of the SC-pulvinar-striatum pathway might be related to orienting and approaching responses, activation of the SC-pulvinar-amygdala pathway induce freezing in mice^7,16,47^. In the cortex and the amygdala, threat-related collicular activity could be integrated with past experience and contextual factors to adaptively modulate the performance of defensive behaviors. For instance, rapid spatial learning of the location of a refuge determines the direction of escape, a response that can switch to freeze if the access to refuge is restricted^48^. Efferences from the central nucleus of the amygdala (CeA) converge with those of the dmSC upon the PAG, where these efferences can directly modulate defensive responses.

By performing simultaneous deposits of fluorescent tracer in the CeA and SC, Usunoff et al.^35^ demonstrated that the same PBG cells project to the contralateral SC and the contralateral amygdala in the rat. This result indicates that the same visual activity elicited by aerial visual field stimulation is simultaneously propagated by the aPBG to the contralateral SC and CeA, which further relates the aPBG division with fear responses.

In addition to the excitatory projection originated by parvalbumin + SC cells, the PBG also receives inhibitory projections from a population composed by stellate, narrow field and horizontal GABA-ergic cells, located in the SGS layer of the SC^40,49^. At present the physiological and functional role of this projection has not been investigated; however there is evidence that local inhibitory SGS neurons play an important role in tuning the physiological responses to aversive visual stimuli at the SC level^50^.

### PBG mediated binocular interactions in the SC

The fact that the contralateral PBG projection to the SC corresponds to the binocular representation of the aerial visual field links the PBG to the SC role in controlling escape reactions. Direct PBG-SC interactions may add a bottom-up control to the rapid implementation of defensive behaviors, possibly by enhancing neural responses of looming sensitive neurons, especially TGCs in the medial SC. Binocular summation in this portion of the visual field would bring an increased sensitivity to moving stimuli that may facilitate aerial vigilance. In turn, enhanced activity transmitted to the deeper layers could raise the dmSC firing above the PAG threshold to initiate escape^37^. At the same time, enhanced TGC responses would be transmitted to the lateral amygdala via the SC-pulvinar pathway, and to the CeA, via the LA-CeA connections, where they could interact with the direct PBG afferences.

The crossed PBG-SC projection constitutes the main source of binocular interaction in the superficial SC, adding a binocular function to the repertoire of visual operations of the PBG. Graybiel^51^ first described in detail the topography of the parabigeminal terminals in the SC of cats, stressing that the contralateral projection covers the rostral half of the collicular surface where the area centralis and the frontal binocular field are represented, suggesting a link between the PBG and binocular operations. Notably, the PBG-mediated binocular operations taking place in the overhead binocular field of rodents would depart from those mediating stereoscopic vision, as normally attributed to the frontal binocular field in most mammals. The physiology of stereopsis depends on combining activity from both retinas to evaluate image disparity. In mammals this is attained by the segregated arrangement of ipsilateral and contralateral retinal projections in the lateral geniculate nucleus in the thalamus that are subsequently integrated in the visual cortex^52,53^. However, the ventral retina that represents the overhead visual field lacks ipsilateral central projections in *O. degus*^32^. In the case of the SC, like in most rodent species reported, the ipsilateral projection is mostly limited to the rostral portion, representing the frontal visual field^32,54–57^. Thus, the PBG contralateral projection to the SC, aside from including this rostral region, provides the main ipsilateral retinal contribution to the medial SC, generating an overhead binocular representation.

Eye tracking measurements in freely moving rats and mice demonstrate that eye saccades and ocular stabilization reflexes combine to stabilize the overhead binocular field, further revealing its relevance in rodent vigilant behavior^9,58^. In addition, successful stereoscopic discrimination of in depth surfaces depends on the alignment of the eyes in the binocular portions of the visual space in mice^59^. We propose that the PBG contralateral projection contributes to keep in register the binocular superposition in the medial SC, in addition to increasing the neural responses when this superposition is attained. Both conditions would be crucial to provoke an oriented and opportune escape response, in which both the motion trajectory and the distance of an approaching threat are consensually estimated from activity in both colliculi. In the same vein, classic work in amphibians have described reciprocal connections between the isthmic nucleus (NI) and the optic tectum (TeO) that match the organization of the PBG-SC connections, i.e. the contralateral isthmo-tectal projection creates a representation of the binocular crescent of one eye in the TeO ipsilateral to that eye^18,60,61^. In physiological experiments the ablation of the amphibian NI completely abolishes binocular responses in the contralateral tectum, without affecting distance estimation in prey catching maneuvers^62^. Thus, comparative data seem to support the idea that the isthmi/PBG-mediated SC binocularity is not linked to depth perception of prey targets, and thus, the possibility that it might be related to range estimation of an approaching predator deserves further investigation at the behavioral and neuronal level. Moreover, in view of this evidence, the physiology of binocular vision at the tectal level and its possible dependence on PBG projections demands a careful experimental research that at present is completely absent.

### The ipsilateral parabigemino-tectal pathway

Up to now, no attempt to selectively stimulate different divisions of the nucleus has been made^16,17^. Therefore, the possibility that the ipsilateral PBG-SC projection departs in its function from the contralateral and amygdalar projection demands further research. Comparative evidence suggests a possible role for the ipsilateral projection. In all groups of vertebrates there are ipsilateral isthmo-tectal reciprocal connections, whereas a contralateral isthmo-tectal projection seems to be more restricted to fishes^63^, amphibians and mammals^18^. In the case of rodents, these ipsilateral connections may be equivalent to those reported in birds between the TeO and the nucleus isthmi parvocelullaris (Ipc)^64–67^. The avian Ipc controls, in a space specific manner, the ascending propagation of retinal activity to higher visual areas, implementing a stimulus selection mechanism perhaps involved in spatial attention^68–70^. The conserved character of this network suggests that the pPBG ipsilateral projection may serve a similar role in spatial attention^5^. As this projection encloses the complete medio-lateral extent of the SC, the pPBG role in approaching or aversive responses could depend on which SC region is being modulated. Physiological and behavioral data indicate that the PBG does play a role in orienting to and pursuit of small visual targets. In the awake cat, the firing rate of PBG cells encodes for retinal position error, the distance formed between gaze direction and the location of the pursued target^18,71^. In addition, a recent report showed that the chemogenetic inactivation of narrow field cells in the SC diminishes orienting, pursuing and capture maneuvers in hunting mice^72^, while the inactivation of parvalbumin-positive cells that mediate escape responses^17^ does not affect hunting behavior^72^. Since both cell types project to the PBG it would be interesting to find out whether they do it to different subdivisions.

In conclusion, we have shown in *O. degus* that the aPBG projects to the contralateral medial SC, allowing binocular interactions in the SC area representing the aerial binocular field. This topology links the mechanisms by which the SC and the PBG produce defensive behaviors (Fig. 10). The ipsilaterally projecting pPBG could be related to the complementary behaviors of orienting and approaching to a prey, also proposed for the PBG.

## Methods

### Animals

A total of 25 *Octodon degus* from both sexes, weighting 180–220, where used in this study. The animals were captured in the wild and maintained in an animal facility at the Universidad de Chile. The individuals that received single tracer injections in the SC were also part of a previous study from our laboratory^42^. All animals were treated following the protocols established by the Comité Institucional de Cuidado y Uso Animal (CICUA) of the Universidad de Chile and the National Institute of Health Guide for the Care and Use of Laboratory animals, and the guidelines of the Animal Ethics Committee of the University of Chile. All experimental procedures were approved by the Faculty of Sciences of University of Chile Ethics Committee.

### Surgery and tracer injections

A total of twelve *O. degus* received neuroanatomical tracer injections. The surgical procedure was performed as we previously described in Deichler et al.^42^. To induce sedation, *O. degus* were placed in a gas anesthesia induction chamber with 1.5–2% isofluorane in medical oxygen, delivered at a rate of 100 ml/kg/min. Once unconscious, individuals received an intraperitoneal injection of diazepam (5 mg/kg) and were returned to the induction chamber for additional 5 min. Then, the animals were transferred to a stereotaxic apparatus and prepared for sterile surgery. Anesthesia was maintained by supplementing isofluorane 2% at a flow rate of 50 mL/kg/min through a modified silicone mask adapted to the orofacial anatomy of degus. Animals were connected to a thermoregulated heating blanket, and 1 ml of saline solution, injected subcutaneously, and ophthalmic gel applied to the eye were used to prevent general and corneal dehydration.

The posterior aspect of the skull was exposed by means of a skin incision and, depending on the experiment, a craniotomy was performed to expose either the cortex overlaying the SC or the PBG. A glass pipette (tip diameter 15–20 μm) containing CTB (List Biological Laboratories, Campbell, CA; 1% in phosphate buffer 0.1 M) was lowered vertically into the SGS layer of the SC or into the PBG, depending on the case. Collicular CTB injections were directed toward either the medial or the lateral aspect of the SC. The injections were made by iontophoresis, using 8 μA pulses of positive current, 7 s duty cycle for 20 min, delivered by a Midgard source (Stoelting, Wood Dale, IL), supplemented with one or two pressure pulses applied by a Picospritzer (Science Products GmbH, Germany). Finally, the incision was closed and the animals received an additional injection of 3 ml of saline solution in combination with analgesics (Carprofen 5 mg/kg).

After a 3-to-5-day survival period, animals were sedated in the induction chamber, given an overdose of ketamine/xylazine and perfused intracardially with saline followed by 4% paraformaldehyde (PFA). The brains were removed from the skull and postfixed overnight in the perfusion fixative. Then the brains were transferred into a 30% sucrose solution until they sank, sectioned and placed in PBS (0.1 M, pH 7.4) for further immunohistochemical procedures.

### Immunohistochemistry

Immunohistochemical procedures were performed to reveal the expression of the choline acetyltransferase protein (ChAT) and the transport of the neural tracer cholera toxin subunit b (CTB) as reported in Deichler et al.^42^. All incubation steps were performed at room temperature in gentle agitation and preceded by PBS washes (3 × 5 min). First, to quench endogenous peroxidase activity, brain sections were incubated in 0.3% H2O2 and 10% methanol in PBS for 10 min. Next, the tissue was incubated overnight at 4 °C with a primary antibody (1:20000 for anti-CTB [List biological Laboratories, RRID: AB_10013220] and 1:1000 for anti-ChAT [Millipore, RRID: AB_90661]) diluted in a blocking solution containing 0.3% Triton X-100 and 3% normal donkey serum (Coning, CA# 35-030-CV, Lot# 1512126) in PBS. Then they were incubated for 2 hours with a secondary antibody (anti-goat, Jackson ImmunoResearch Labs, CA# 705-066-147, Lot# 132567) diluted 1:2000 in blocking solution. This step was followed by a 2-h incubation in avidin–biotin complex (Vector Laboratories, CA# PK6100, Lot# ZC 1003, ZC 1004) diluted 1:500 in 0.3% Triton PBS. Finally, to initiate a DAB-peroxidase reaction, sections were incubated in a solution of 0.25 mg/ml diaminobenzidine hydrochloride, 0.1 M NiCl_2_, 0.001% H_2_O_2_ in PBS. One of the four series of sections reacted for CTB was counterstained with Giemsa to reveal the cells bodies.

### In situ hybridization

RNA probes were designed using the *Octodon degus* nucleotide databases (NCBI Nucleotide, RRID:nlx_84100) and the alignment tools of the NCBI website (NCBI BLAST, RRID:nlx_84530; Primer-BLAST, RRID:OMICS_02343; https://www.ncbi.nlm.nih.gov). To amplify the cDNA corresponding to each probe, specific pairs of primers (Table 1) were designed and commercially synthetized (IDT DNA, Coralville, IA). Each of the amplified transcripts was located in the coding region of their corresponding mRNA.

**Table 1:** Sequences of PCR primers used to amplify specific cDNA sequences of each marker.

	Forward primer	Reverse primer	Fragment (bp)
ChAT	5′-CATACCCAGACACGCTGGT-3’	5′-TGGCACCATTCTGGCTGTAG-3’	403
VAChT	5′-CCCTTTTCGCATTCGCTGAG-3’	5′ -TGAGGTAGACGCCCAAAACG-3’	534
VGluT2	5′-CACTAAGTCGTACGGTGCCA-3’	5′ -TGGTGATGCATTCTAGCGCC-3’	247

Digoxigenin-labeled riboprobes specific for VGluT2, VAChT and ChAT were synthetized following the protocol previously implemented in our laboratory and described in González-Cabrera et al.^19^. To perform the in situ hybridizations, nine degus were perfused intracardially with saline solution followed by PFA 4%. Their brains were then removed and postfixed for 12 h in PFA 4%. The brains were then cryoprotected with 30% sucrose solution and cut on a freezing microtome at 60 µm. Alternative series were used to assess the expression of each mRNA probe. Free-floating sections were washed twice in PBS (0.01 M phosphate buffer pH 7.4; 0.02% NaCl in DEPC-treated water) followed by 10 min of incubation in acetylation solution (625 μl triethanolamine; 88 μl HCl; 125 μl acetic anhydride in 50 ml distilled water). The sections were incubated in proteinase K/PBST solution (Proteinase K 10 μg/ml, Promega) for 10 min at 37 °C and then post-fixed in PFA 4% at room temperature for 20 min, washed three times with PBST, and prehybridized in hybridization buffer for 3 h at 65 °C (50% formamide, Merck, Darmstadt, Germany; 1.3X standard saline citrate [SSC], pH 5.3, Winkler, Santiago, Chile; 5 mM EDTA, Winkler; 200 lg/ml tRNA from salmon sperm; 0.002% Tween-20; 0.005% CHAPS, Calbiochem, La Jolla, CA; 100 lg/ml heparin, Calbiochem). Then, the sections were incubated in a new hybridization buffer containing the specific RNA probes (30–60 ng/ml) for 16–18 h at 57 °C. Next, the sections were washed twice for 30 min at 57 °C in solution A (5X SSC, pH 5.3; 50% form- amide; 1% sodium dodecyl sulfate [SDS]) followed by three washes for 30 min at 57 °C in solution B (2.5X SSC, pH 5.3; 50% formamide; 1% Tween-20). Two washes in maleic acid buffer solution (MABT; 100 mM maleic acid, Sigma, St. Louis, MO; 150 mM NaCl; 0.1% Tween-20), were followed by incubation of the brain sections in blocking solution (2% Blocking Reagent, Roche, Indianapolis, IN; 2% heat-inactivated normal goat serum, in MABT) for 2–3 h at room temperature and then incubation for 16–20 h at 4 °C with anti-digoxigenin-AP Fab fragments (1:1000 dilution in MABT; Roche Diagnostics; RRID:AB_514497). Finally, the sections were washed six times in MABT, incubated in alkaline reaction buffer (100 mM Tris, pH 9.5; 50 mM MgCl2; 100 mM NaCl; 1% Tween-20), and developed at 37 °C in the dark by adding NBT/BCIP reagent (NBT 375 μg/ml; BCIP 188 μg/ml; Stock Solution, Roche, Mannheim, Germany).

#### Combined IF/FISH

Fluorescent in situ hybridizations were performed using the Tyramide amplification method (TSA) following the protocol described by Krabichler et al.^73^. At the beginning of the procedure, sections were incubated in a 3% hydrogene peroxide solution in 10% methanol, to inactivate endogenous peroxide. Also, after washing the sections with A and B solutions, two additional 5 min washes were performed using TNT (100 mM Tris–HCl pH 7.5, 150 mM NaCl, 0.1% Tween 20 in filtered H_2_O). Then, sections were incubated at room temperature for 3 h in blocking solution (Blocking Buffer Reagent 1%, Heat Inactivated Horse Serum (HINHS) 1% in TNT). Finally, sections were incubated overnight in a new blocking solution containing the anti-Digoxigenin-POD, Fab fragment (Roche) (1/300) at 4ºC. The following day, sections were washed during 30 min at room temperature in TNT, followed by a 10 min wash in 0.05 M Borate Buffer, pH 8.5. Then, sections were incubated for 1 h, at room temperature in darkness, in a Biotin-tyramide (IRIS Biotech GmbH, Marktredwitz, Germany) solution (0.001% Biotin-tyramide and 0.0015% H2O2 in 0.05 M Borate, pH 8.5). After 3 washes in PBS, fluorescent label was obtained by 2-h incubation with Streptavidin-Alexa-546 (1/500) in PBS and 0,25% Tween 20.

IHC and FISH combined in the same sections was obtained by performing first the FISH protocol to continue with the immunoreaction as described above.

### Projection of retinal retrogradely-filled neurons into the visual field

Processing of the retinas of SC double-injected animals was performed as described in Vega-Zuniga et al.^32^. Following fixation, marks were made with micro-sutures onto the palpebral fissures and dorsal sclera of the eyeballs for orientation. Then, the eyes were enucleated and washed in 0.1 M PBS. Retinas were carefully dissected from their underlying pigmented layer and the optic nerves were severed just beneath their retinal attachment. The isolated retinas were mounted with Fluorsave (EMD Millipore Corporation, Temecula, CA) mounting medium, coverslipped and then scanned using a confocal microscope (Zeiss LSM 770). Whole mounted retinae, containing labeled RGCs after CTB injections in different parts of the SC, were reconstructed into a standard spherical retinal space using the Retistruct package in R^74^. Then, using the same program, an orthographic projection was generated in order to visualize toward which part of the visual space the labeled neurons were looking. A representation of the visual field of the degus was elaborated using a customized program written in Matlab by Symonova and Vega-Zuniga with campimetric data from Vega-Zuniga et al.^32^**.**

### Soma size estimations and statistical analysis

To estimate the somatic volume of CTB-retrogradely-labeled neurons in the PBG, we measured their cross sectional area using the nucleator probe (isotropic nucleator, four rays) of the StereoInvestigator (MBF Bioscience). To compare the somatic volume (µm^3^) between contra and ipsilateral labeled cells, we performed a repeated measure analysis of variance (ANOVA) after logarithmic transformation of the data due to the violation of normal distribution^75^ (Kolmogorov–Smirnov Normality test: *D* = 0.146, *p* = 2.193 × 10^–13^) and homocedasticity (Fligner-Killeen test of homogeneity of variances: *FK* = 47.08, *df* = 1, *p* = 6.823 × 10^–12^). Statistical analysis was performed using software R (R Foundation for Statistical Computing, Vienna, Austria 2009).

### Behavioral tests

Based on the experimental designs of De Franceschi et al.^8^ and Yilmaz and Meister^10^, we constructed an experimental arena 48 cm wide × 35 cm deep × 30 cm high, containing two computer monitors, one on the roof for aerial stimulus display, and the other on one side of the box for frontal and lateral stimulus display. A wooden refuge was placed on the side of the floor facing the side monitor. The stimulus was presented in one monitor when the animal was freely moving near the center of the arena with both monitors in the field of view. The stimuli were generated using the open-source software Psychopy in Python^76^. *O. degus* movements were video recorded with a camera, using VCL Media Player (30 frames/s) and tracked with the software Icy, an open bioimage informatics platform^77^. Analyses were made using a Python custom-made routine. Statistics were performed using software R. Instantaneous speed was calculated as the trajectory covered by the animal between two consecutive frames, pixels/frame were converted into cm/s. To assess a significant change in speed under visual stimulation, we calculated the 95% confidence interval of speeds obtained from 3 s of spontaneous movements before visual stimulation (baseline). The average of instantaneous speed across animals was calculated and compared with the baseline statistics.

The looming stimulus consisted of a 2° black disk rapidly widening to 50° in a 250-ms lapse. Stimuli were presented randomly to each individual (*n* = 19). The degus were habituated to the box for 15 min. the day before the first trial. Each animal was stimulated only once with a given stimulus in single trials at least three days apart.

## Supplementary information

Supplementary figures.
