# Supplementary material for: A specialized reciprocal connectivity suggests a link between the mechanisms by which the superior colliculus and parabigeminal nucleus produce defensive behaviors in rodents
Source: Sci Rep. 2020 Oct 1;10:16220. doi: 10.1038/s41598-020-72848-0 (PMC7530999; doi:10.1038/s41598-020-72848-0)
Supplement: Supplementary file 1 — Supplementary figures. [file 41598_2020_72848_MOESM1_ESM.pdf]

Supplemental Information

A specialized reciprocal connectivity suggest a link between the mechanisms by which the Superior Colliculus and Parabigeminal nucleus produce defensive behaviors in Rodents

Alfonso Deichler, Denisse Carrasco, Luciana Lopez-Jury, Tomas Vega-Zuniga, Natalia Márquez, Jorge Mpodozis and Gonzalo J. Marín

Supplemental Figures

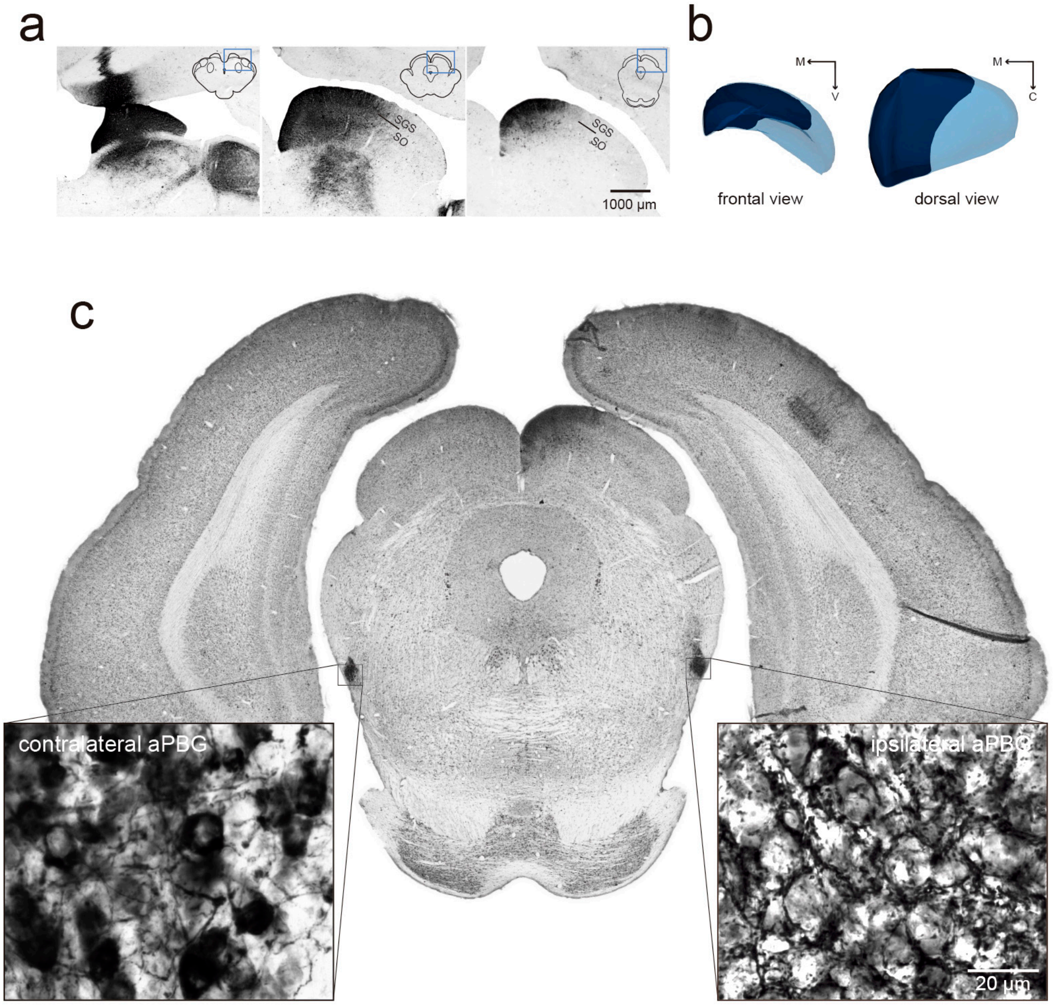

Figure S1. Projection of the medial SC upon the aPBG. A representative case of a CTB injection that covered the complete extent of the medial SC. Panel (a) Injection site at caudal, intermediate and rostral collicular levels. (b) 3D reconstruction of the injection in the superficial SC. (c) Anterograde labeled terminals cover the ipsilateral aPBG and retrogradely labeled cells are observed in the contralateral aPBG. Note the nest of terminals upon the empty spaces left by unlabeled cell bodies in the ipsilateral aPBG.

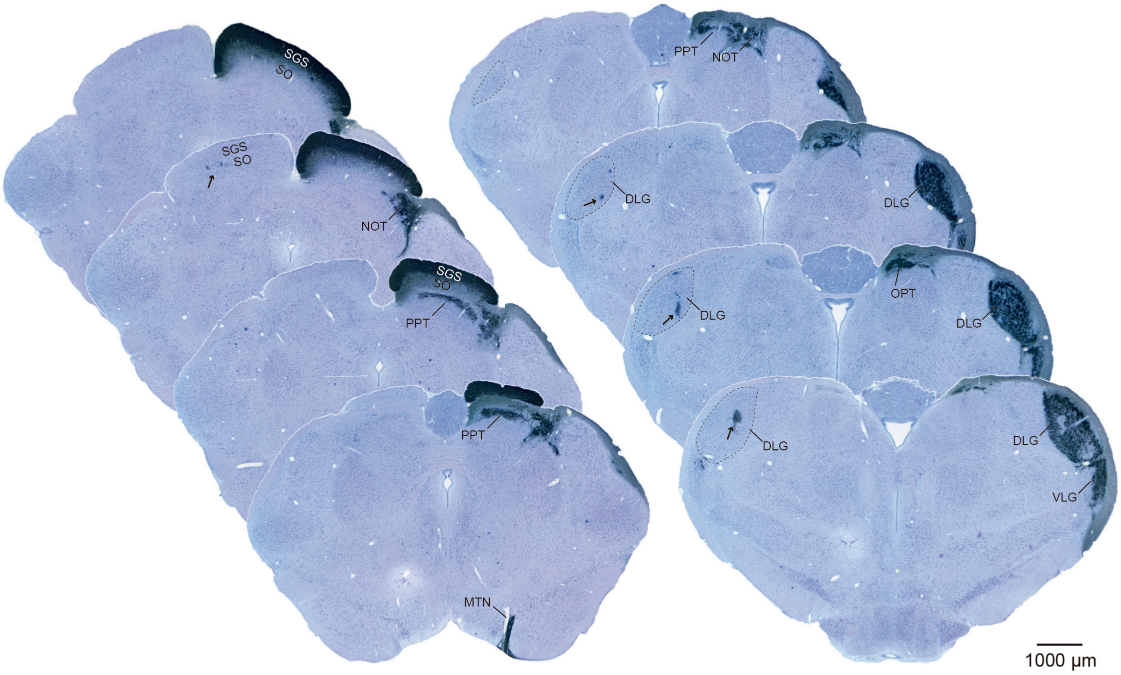

Figure S2. Central retinal projections in *O. degus*. A series of coronal sections showing labeled central targets after a monocular injection of CTB. The ipsilateral side to the injected eye is displayed to the left. Note that the projection to the SC is almost completely decussated, with only a small patch of ipsilateral fibers at mid rostral SC level (arrow in the second section at the left), while a more conspicuous but still reduced patch of retinal projections is observed in the ipsilateral dorsal lateral geniculate (arrows in the sections containing the DLG at the right; Data obtained from previous work in our laboratory[32].) DLG, dorsal lateral geniculate nucleus; MTN, medial terminal nucleus; NOT, nucleus of the optic tract; OPT, olivar pretectal nucleus; PPT, posterior pretectal nucleus; SGS, stratum griseum superficiale; SO, stratum opticum; VLG, ventral lateral geniculate nucleus.

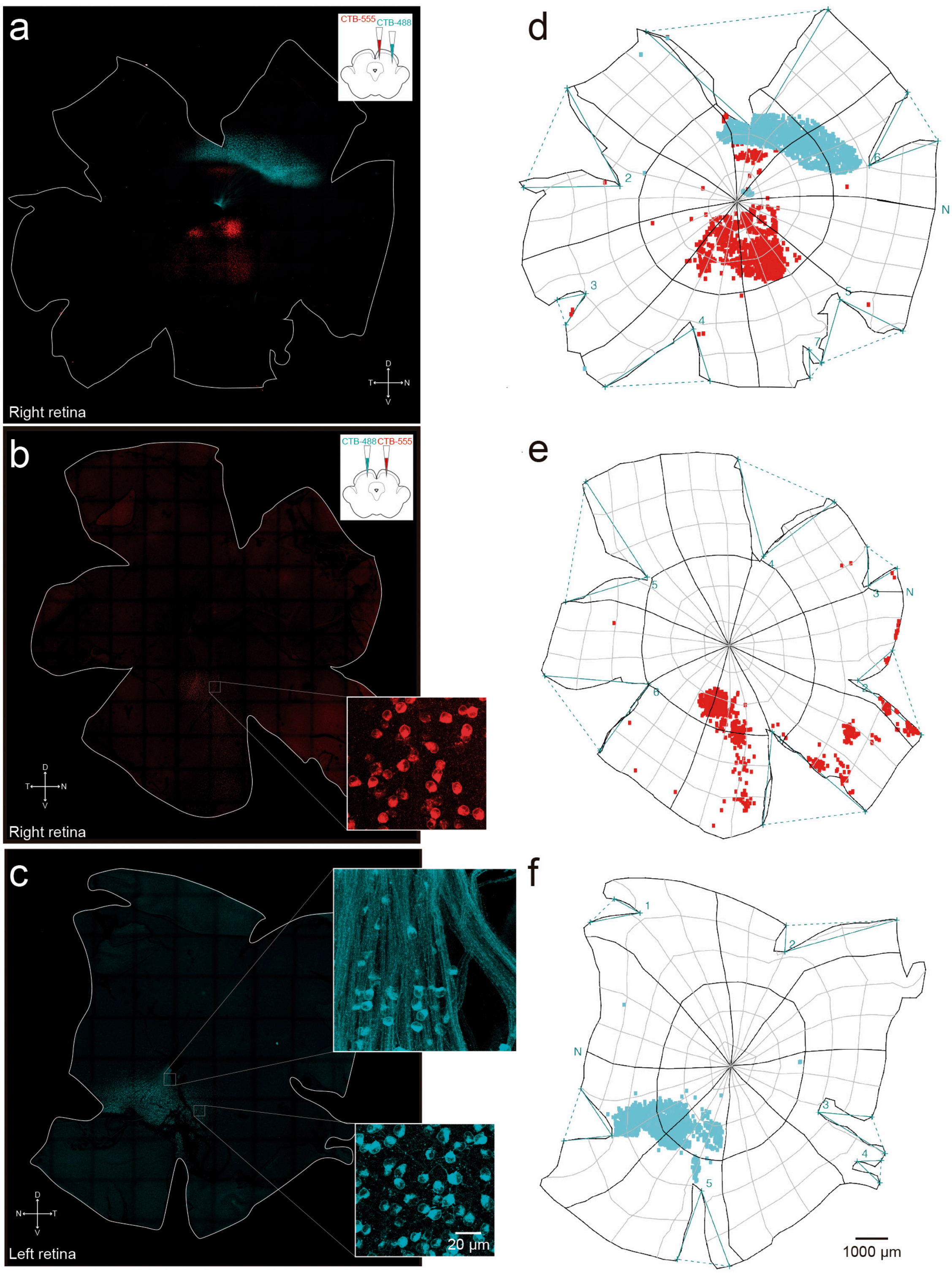

a
CTB-555
CTB-488
Right retina
b
CTB-488
CTB-555
Right retina
c
Left retina
20 µm
d
e
f
N
1000 µm

Figure S3. Retrograde retinal labeling and reconstructed retinas after CTB injections in the SC. (a) and (b, c) correspond to cases presented in figures 5 and 6, respectively. The nasal (N), dorsal (D), temporal (T) and ventral (V) poles of the retina are indicated. Note that the distribution of RGCs follows a topographic array, with the ventral and dorsal retina labeled after medial and lateral SC injections, respectively. Also note the absence of ipsilateral labeling after CTB deposits in the medial SC (b, c, e, f). (d – f) retinal outlines with the marked nasal pole (N), cuts marked up and polar coordinates generated in Retistruct[74]. Dark cyan lines connect the vertices with the apex of the each cut. Dots represent individual ganglion cells labeled by each fluorescent tracer.

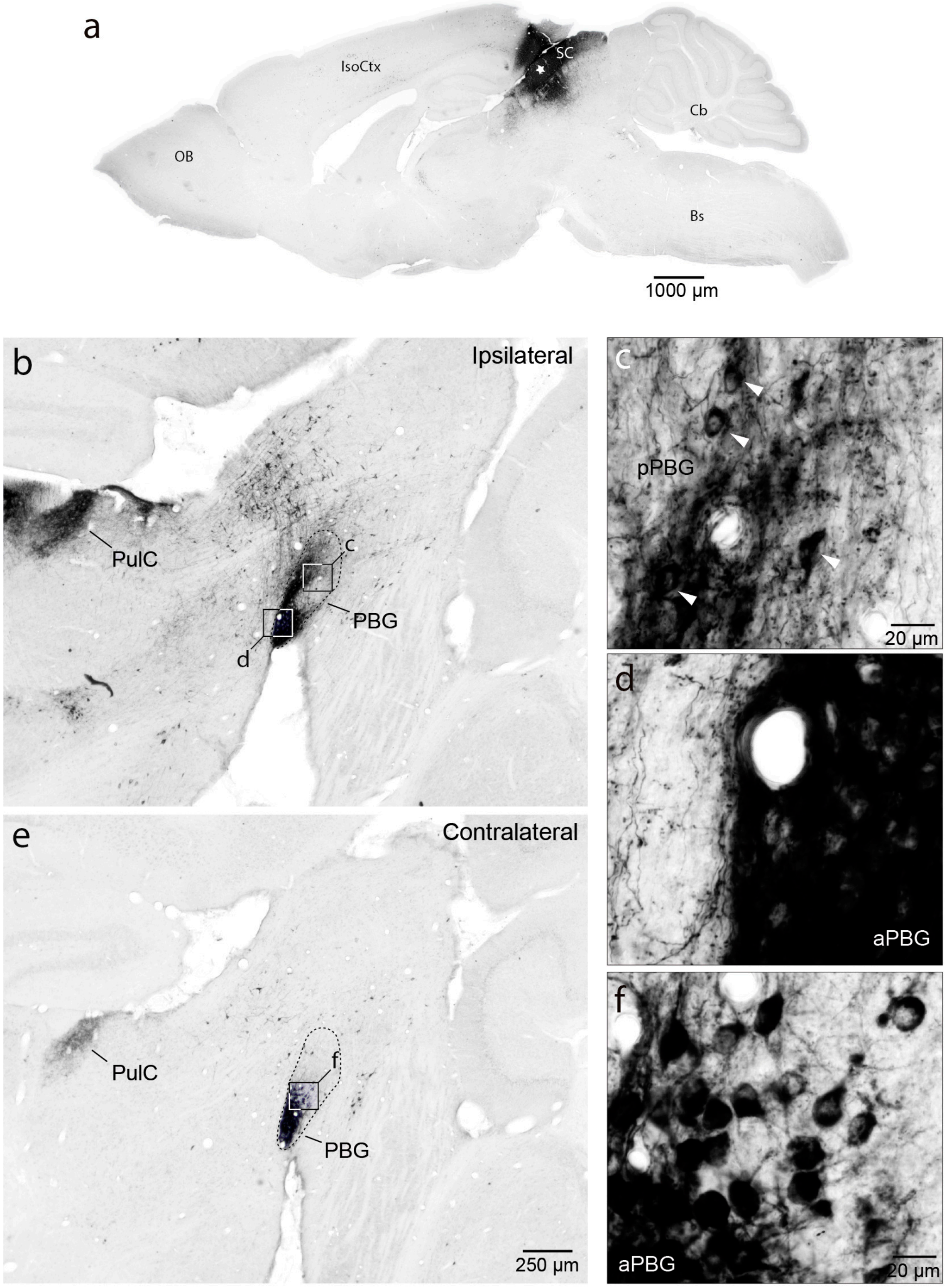

a
IsoCtx
SC
Cb
OB
Bs
1000 μm
b
Ipsilateral
PulC
c
PBG
d
c
pPBG
20 μm
d
aPBG
e
Contralateral
PulC
f
PBG
250 μm
f
aPBG
20 μm

Figure S4. Bilateral parabigemino-tectal projections in the mouse. (a) Sagittal section showing a CTB injection in the SC (a representative case from an $n = 3$). As in degus, retrogradely labeled cells are found in the ipsilateral posterior PBG (b, arrowheads in c) and in the contralateral anterior PBG (e, f). Also note that the CTB deposit is localized in the anterior-medial SC (asterisk in a), producing a dense plexus of anterogradelly labeled terminals in the ipsilateral aPBG, similar to what we found in degus (b, d).

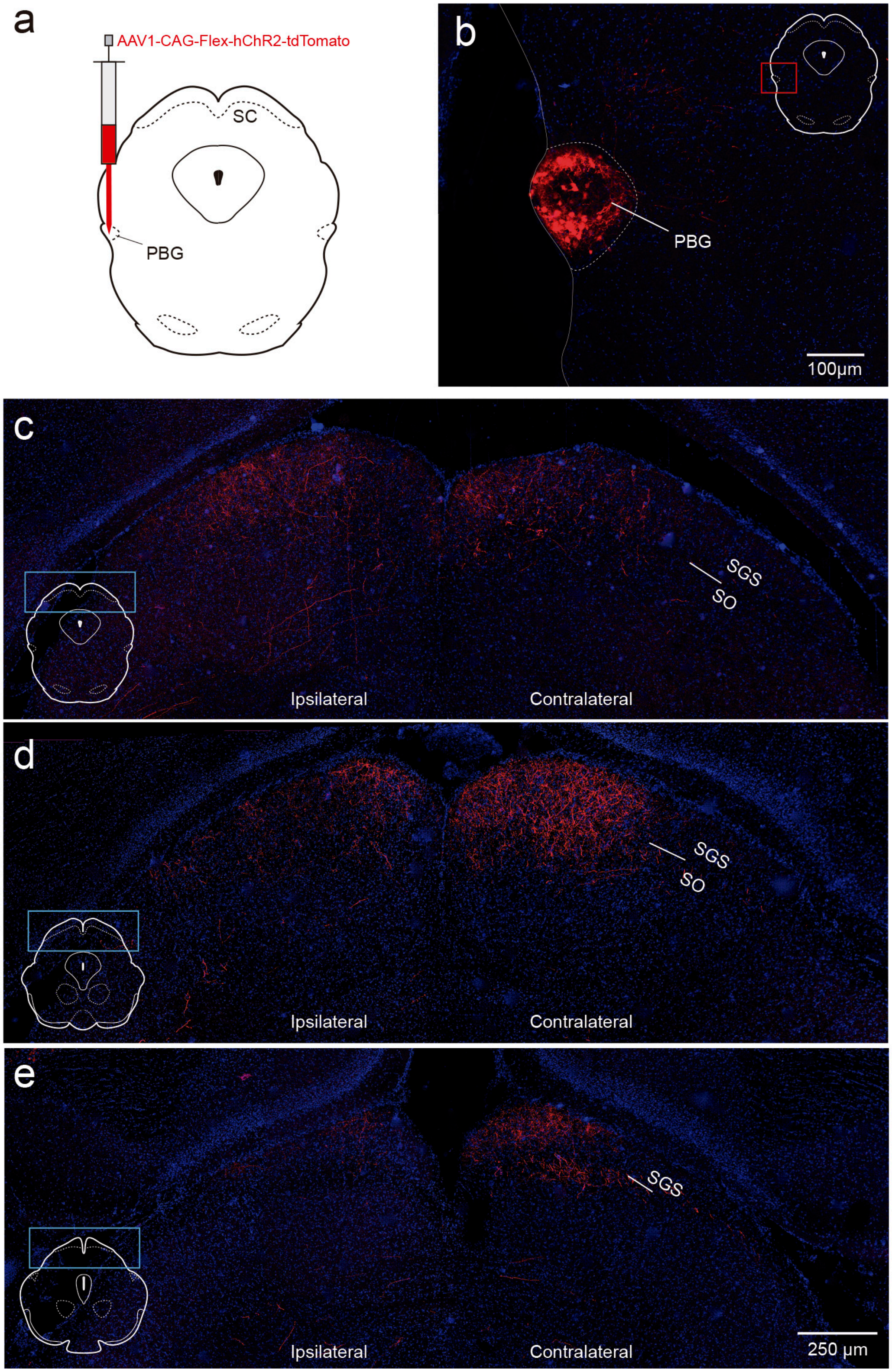
a
AAV1-CAG-Flex-hChR2-tdTomato
SC
PBG
b
PBG
100μm
c
SGS
SO
Ipsilateral
Contralateral
d
SGS
SO
Ipsilateral
Contralateral
e
SGS
Ipsilateral
Contralateral
250 μm

Figure S5. Topography of PBG axonal terminals in the superficial SC of the mouse. To label the PBG axons in the SC, we used a transgenic mouse line that expresses the Cre recombinase gene associated to the Chat promoter (ChAT-IRES-Cre mice, Jackson Laboratories stock #006410). Injection of a Cre-dependent adeno associated virus (AAV1-CAG-Flex-ChR2-tdTomato) in the PBG of these mice (n = 4, Schematics in (a)), allowed the selective targeting of PBG neurons (b). Terminals expressing tdTomato were found across the whole rostral-caudal extent of both colliculi (c – e). As in the degus, axonal terminals covered the medial-lateral extent of the ipsilateral SC, with a medial emphasis in this case because of the rostral bias of the injection, while in the contralateral side they were restricted to the medial collicular aspect.
